# Ceramic veneers on central incisors without finish line using bopt in a case with gingival asymmetry

**DOI:** 10.4317/jced.55688

**Published:** 2019-06-01

**Authors:** Helia Peris, Laura Godoy, Pablo G. Cogolludo, Alberto Ferreiroa

**Affiliations:** 1 Professors of the master of prosthesis, implant prosthesis and aesthetics of the European University of Madrid

## Abstract

**Objectives:**

This clinical case report describes the rehabilitation of central incisors with veneers in a patient with gingival asymmetry. The teeth were prepared without finish line, applying BOPT concepts to correct asymmetry, and obtained a harmoniously integrated restoration with optimal periodontal health.

**Clinical implications:**

Biologically oriented preparation technique (vertical or “feather edge” preparation) was used to obtain gingival symmetry. Teeth were prepared without horizontal finish line to achieve correct emergence, soft tissue adaptation and stabilization, while maintaining biological space, both at the provisional restoration stage and later when definitive restorations were placed. To perform the technique correctly, it is essential to perform adequate periodontal diagnosis to verify the space available between the bone crest and the future margin of the restoration.

**Conclusions:**

It is possible to correct gingival asymmetry by performing dental preparation without finish line providing a correct periodontal analysis is first performed, which will contribute to successful soft tissue stabilization.

**Clinical Significance:**

Beyond of all the prosthodontic preparation techniques, knowledge of B.O.P.T. (Biological Oriented Preparation Technique) allows us to achieve predictable and consistent results in terms of periodontal health and gingiva architecture surrounding ceramic veneers. Nevertheless long term studies are necessary to ensure the benefits of this techniques.

** Key words:**Gingival margin, emergence profile, biologically oriented preparation technique.

## Introduction

The generalized demand for optimal esthetics in dental treatments involving prosthetic restorations can only be met through a detailed consideration of prosthetic, periodontal and esthetic factors. To ensure predictability and a successful outcome, it is important to assess both gingival esthetics, dental proportions, dental composition and axial inclination (1,2). When restorations are cemented in anterior regions using conventional techniques, the symmetry of the resulting restoration will be determined by the individual clinician’s dental preparation skills and the laboratory technicians prosthetic fabrication skills (3,4). As an alternative to conventional treatment, the use of Biologically Oriented Preparation Technique (BOPT) for applying ceramic veneers in the anterior regions facilitates periodontal tissue management and symmetry. Teeth are prepared without a horizontal finish line, which produces a correct emergence, good adaptation and stabilization of soft tissues, and can even correct soft tissue anomalies and asymmetries (5-8).

This case report describes a 30-year-old female patient who wished to change several inadequate restorations that were generating inflammation and gingival alteration.

At the first clinical session, a complete periodontal analysis was performed to determine the biological space available for the restorations. Then BOPT was employed, creating blood coagulate that stabilized around the provisional restorations; six weeks later, soft tissue maturation and stabilization had taken place. A register was taken to fabricate conventional feldspathic ceramic veneers. When the veneers were cemented in place, gingival symmetry and correct integration of the restorations were observed, which were maintained at the one-year follow-up, when gingival health and gingival thickening were seen to have been generated by the new restorations without finish line.

## Case Report

A thirty-year old patient came to the dental clinic at Madrid International University seeking to improve the esthetics of her upper central incisors. The patient presented a low smile line and expressed her dissatisfaction with the esthetics of the upper central incisors.

**Figure 1:**
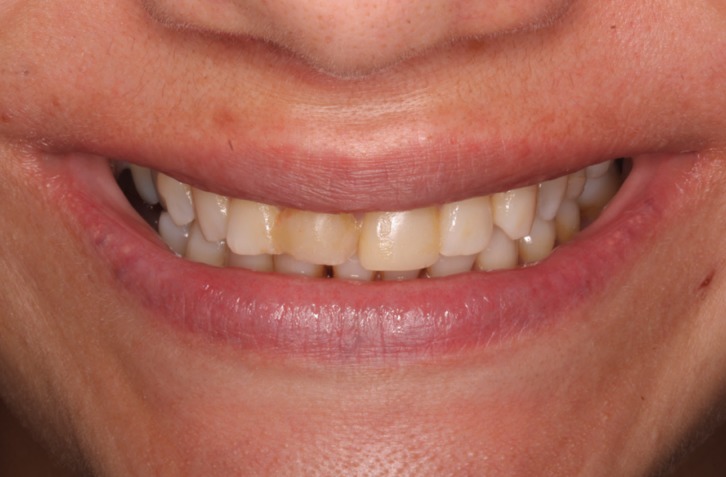
Inicial extraoral picture. The patient presented a low smile line and expressed her dissatisfaction with the esthetics of the upper central incisors.

**Figure 2:**
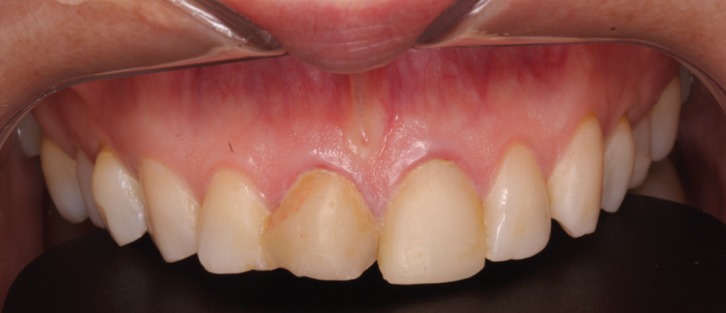
Inicial intraoral picture. The patient presented two composite veneers were discolorated, misshapen and showed defective polishing causing irritatión of the surrounding soft tissues.

In clinical examination, the presence of two composite veneers was observed that were discolored and misshapen. In particular, the edge of restoration on the upper right central incisor was bonded to the upper right lateral incisor. Both reconstructions showed defective polishing particularly in the area of the margin causing irritation of the surrounding soft tissues resulting in inflammation and alteration of gingival symmetry at both gingival zeniths. The axial inclination of the right upper central incisor produced an appearance of distortion due to the mesialization of the corresponding gingival zenith, which contributed to the poor esthetics observed (9). (Figs. 1,2)

**Figure 3:**
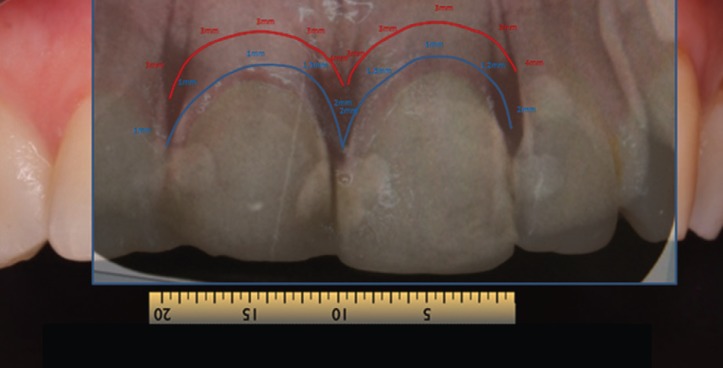
When exploring patient´s periodontal anatomy the parameters that were observe are: In RED the position of the bone crest and in BLUE the epitelial insertion level.

In gingival examination, it could be seen that the gingival zeniths of the upper right central incisor and upper left central incisor lacked a harmonious pattern derived from their apparent asymmetry. To explore the patient’s periodontal anatomy, the following parameters were registered (Fig. 3):

- Intrasulcular probing to assess the epithelial insertion level (BLUE).

- Probing to check the position of the bone crests (RED).

Afterwards, the information registered was checked by radiological exploration performed using a parallelizer.

On the basis of the data obtained, new restorations were planned with the assistance of a Digital Smile Design protocol (10,11) to determine the correct morphology and proportions, and the correct positioning of both incisors and gingival zeniths. This data was transferred to a wax-up which was used to produce a mock-up (12).

**Figure 4:**
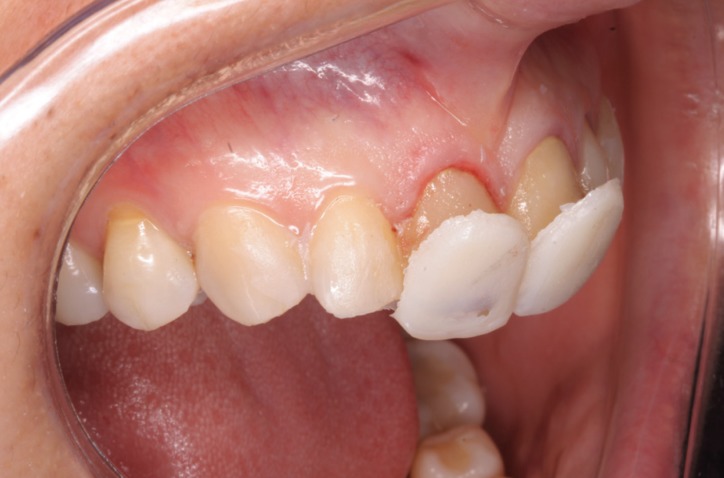
When placed de mock-up on the teeth we see the mark that produces the resin and we observe that it is not necessary to invade the biological width as there was sufficient margin from the gingival margin to the bone crest.

A further appointment was made to apply the mock-up to assess morphology and determine functional registers and the position of the new gingival margin, and to assess the need for periodontal treatment (gingivectomy/crown lengthening). When placed on the teeth, the cervical area was probed and it was seen that it was not necessary to invade the biological width as there was sufficient margin from the gingival margin to the bone crest (3mm), while the restorations only required 1 mm, leaving 2 mm, which eliminated any need for periodontal surgery (13,14) (Fig. 4).

On the basis of the information obtained, a treatment plan was decided, consisting of two feldspathic veneers on the upper central incisors as well as a direct composite restoration on the upper right lateral incisor.

The treatment sequence was as follows.

**Figure 5:**
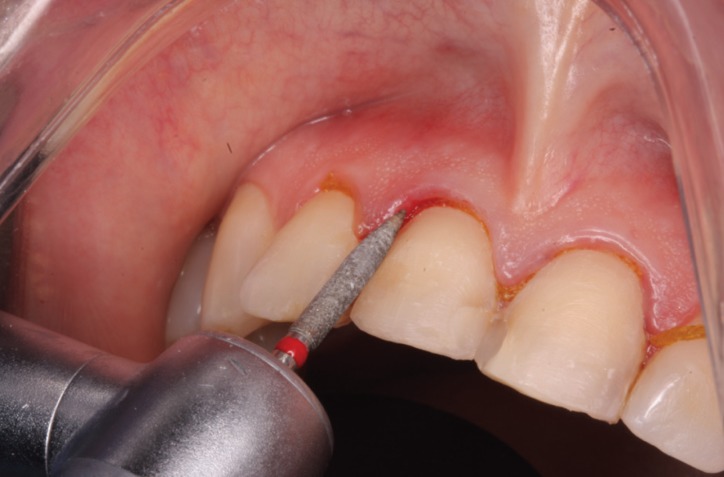
Dental preparation with BOPT technique. The technique aims to create blood coagulate during preparation that later stabilizes when the provisional restorations are placed.

Dental preparation was performed with help from a silicon index taken from the diagnostic wax-up, preparing the teeth without horizontal finish line, respecting biological width and provoking intrasulcular de-epithelialization according to the BOPT procedure proposed by Dr. Ignazio Loi (5). The technique aims to provoke blood coagulate during preparation that later stabilizes when the provisional restorations are placed and matures to form fully structured gingival tissue (Fig. 5).

To fabricate the provisional prostheses, another silicon index was made from the diagnostic wax-up carefully trimming the margins and filling with Bis-Acrylic Protemp 4® resin (3M ESPE, St Paul, MN, USA). When the index was removed and the provisional protheses had set, the margins were checked for adequate definition.

- One fine internal margin corresponding to the intrasulcular part of the prepared tooth.

- One external margin, slightly thicker, corresponding to the shape and position of the gingival margin.

**Figure 6:**
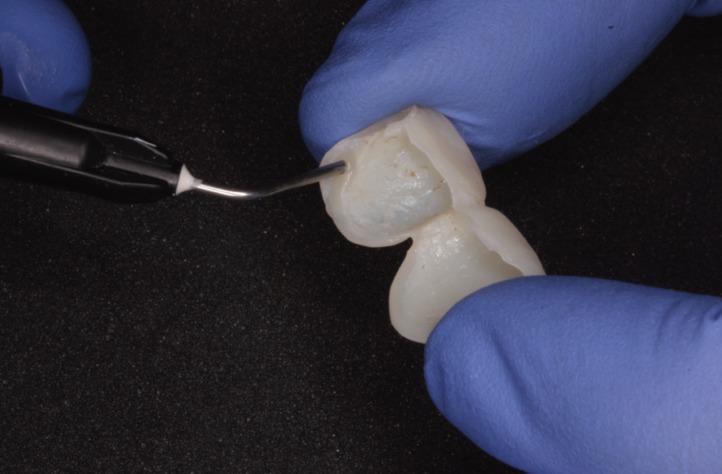
To fabricate the provisional prostheses, the space between the two margins was filled with flow composite, adapting the shape for to reposition the gingival margin to the correct position.

**Figure 7:**
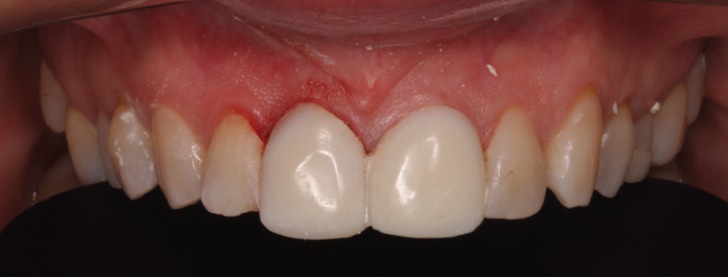
The provisional restoration were placed leaving the soft tissue to mature.

**Figure 8:**
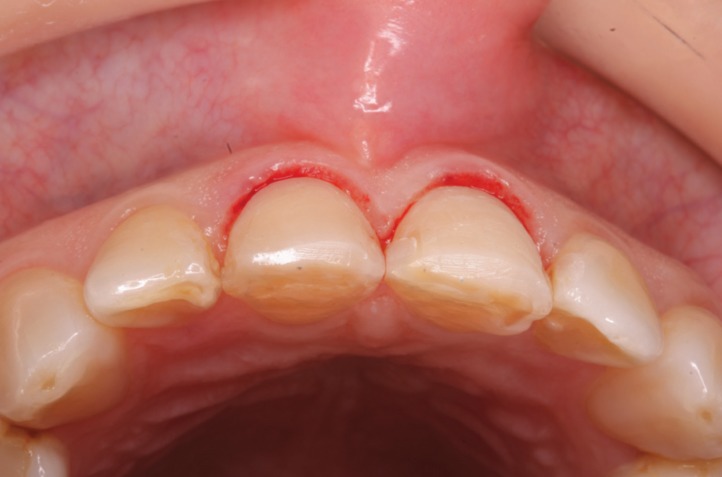
After 4 weeks the soft tissue are mature in the correct position.

To fabricate the provisional prostheses, the space between the two margins was filled with FILTEK® SUPREME XTE FLOW flowable composite (3M Espe, Maplewood, Minnesota, USA), to thicken the margin, adapting the shape of the provisional prostheses so that it would reposition the gingival margin to the correct position determined by DSD, wax-up, and now the provisional restorations (Fig. 6). The provisional restorations were polished with Sof-lex® disks (3M Espe, Maplewood, Minnesota, USA), their fit was checked in the mouth, and they were placed by means of acid etching and adhesive placed on a small vestibular area of the tooth surface leaving the soft tissue to mature (Figs. 7,8).

After 4 weeks, registers were taken using the double cord technique (Ultrapack® #000; Ultradent Products Inc, Köln, Germany) and double mix (Light Body® and Virtual putty®; Ivoclar Vivadent AG, Schaan, Liechtenstein)). To register tooth color, grey scale photographs were taken to determine the tone, and conventional feldspathic veneers were ordered for the right upper central incisor and left upper central incisor.

**Figure 9:**
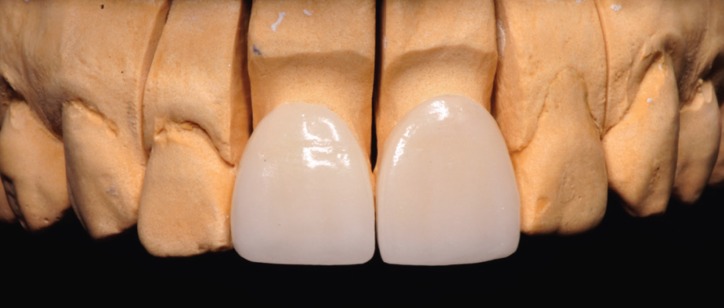
The laboratory technician used the diagnostic wax-up based on DSD to locate the gingival margin.

**Figure 10:**
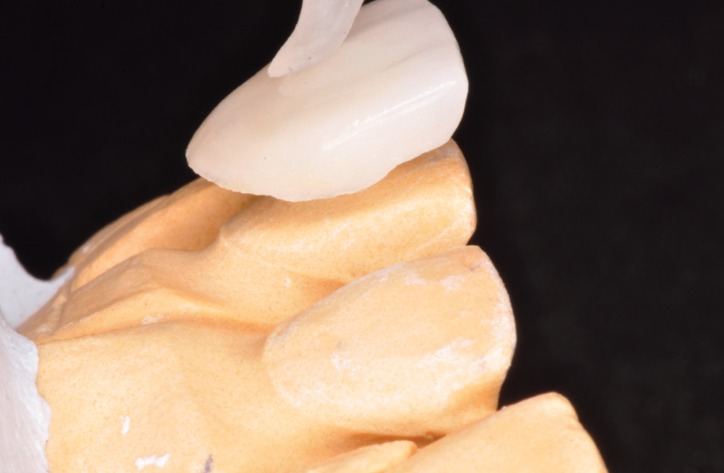
The feldspathic veneers were ordered to improve the aesthetics of the patient.

To fabricate the veneers, the laboratory technician used the diagnistic wax-up based on DSD (created before tooth proparation) to locate the gingival margin (Figs. 9,10).

Once fabricated, the restorations were assessed by two clinicians before being cemented in place.

## Results

**Figure 11:**
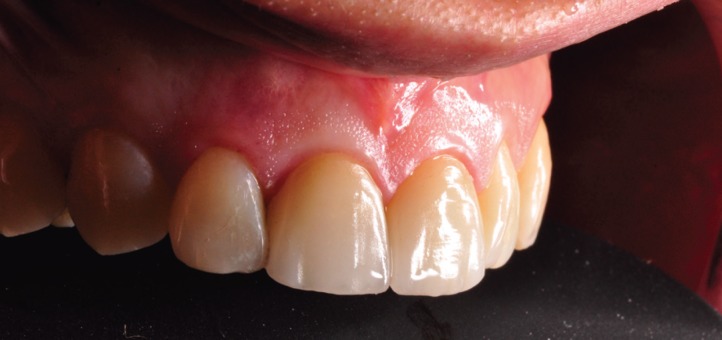
Finally intraoral picture. The ceramic veneers after tooth preparation without finish line, following BOPT concepts.

**Figure 12:**
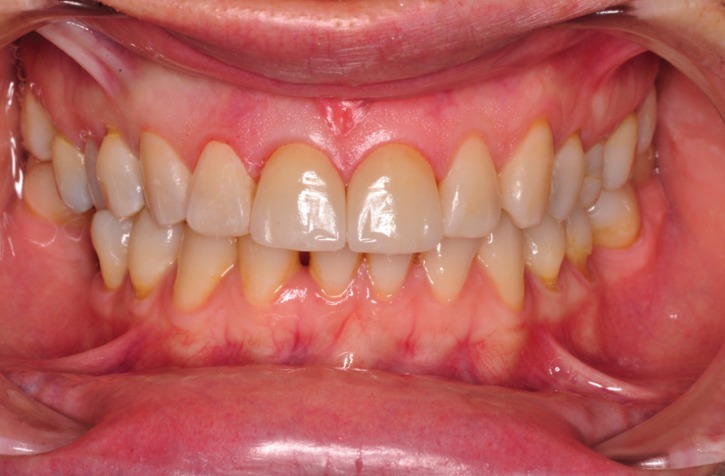
The restorations are harmoniously integrated both esthetically and periodontally. The integration of the restorations was good thanks to correct soft tissue management.

**Figure 13:**
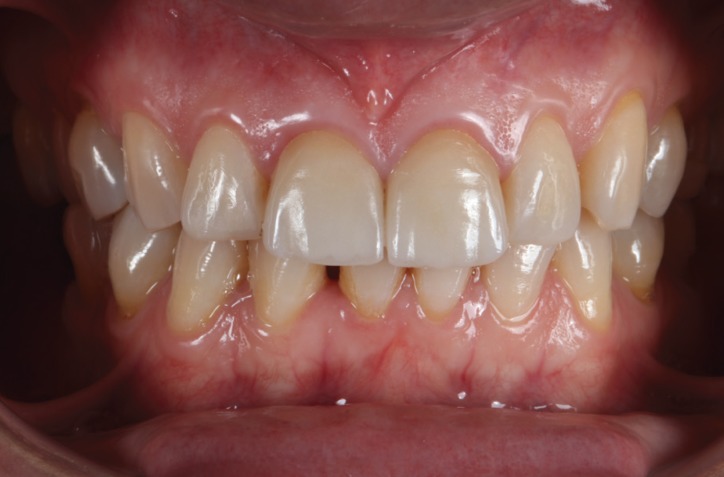
At the one-year follow-up visit, the soft tissues had stabilized correctly, and good gingival health was observed despite the correction of gingival asymmetry.

The application of ceramic veneers after tooth preparation without finish line, following BOPT concepts as proposed by Dr. Loi (5), made it possible to place restorations that were harmoniously integrated both esthetically and periodontally. The chromatic continuity was adequate, the dental proportions, gingival health, the symmetry of the two gingival zeniths were correct and the integration of the restorations was good thanks to correct soft tissue management (15). (Figs. 11,12). At the one-year follow-up visit, the soft tissues had stabilized correctly, and good gingival health was observed despite the correction of gingival asymmetry (5-8) (Fig. 13).

## Discussion

In the present case, biologically oriented preparation technique (BOPT was indicated ) as an alternative to periodontal plastic surgery on the basis of probing and the creation of a mock-up, which showed that it was not necessary to invade biological width; the distance from gingival margin to bone crest was 3 mm while the apical modification required was only 1 mm. The objective of BOPT is to stabilize gingival tissue in the medium and long term maintaining biological width by means of managed invasion of the sulcus during dental preparation. This avoids complications related to conventional horizontal sub-gingival preparation whereby restorations are placed below the gingival margin, which has been shown to be associated with periodontal inflammation and possible gingival displacement (4,16).

BOPT repositions the cementoenamel junction in relation to the prosthetic restoration making it possible to manage dental contours by means of provisional restorations allowing blood coagulate derived from dental preparation to stabilize as mature gingival tissue. This is achieved by shortening or extending the edge of the restoration to reach different levels in the gingival sulcus and so establish the gingival margin, which helps to balance the soft tissues esthetically. Compared with conventional preparation techniques, BOPT is accompanied by greater gingival thickening produced during dental preparation. This reduces the risk of gingival displacement thanks to increased vascularization, regardless of whether the patient presents a thin or thick gingival biotype (5-8).

The disadvantages of BOPT in comparison with horizontal dental preparation derive from its greater complexity and so longer clinical time needed to perform the technique. A new cementoenamel junction must be established despite the lack of dental reference points, which runs a risk – especially when the clinician or technician lacks experience – of uncontrolled invasion of the gingival sulcus. Another disadvantage is related to cementation, as it is not possible to isolate the area precisely as there is no horizontal preparation margin that can be followed, and any excess cement will be difficult to eliminate (6,7). Although the clinical results of BOPT have been promising (5-8), there is not yet sufficient scientific evidence to back its long term prognosis, mainly due to its recent appearance (BOPT was first described in 2013) (5).

The decision to select feldspathic veneers rather than composite was based on the ceramic material’s long-term stability and strength, and its perfectly polished, homogenous surface which helps establish soft tissues without the risk of later inflammation or alterations to soft tissue position or symmetry. In the present case, the substrate was of an adequate color, and all that was required was the modification of dental morphology and the gingival margin, so feldspathic porcelain provided excellent esthetics despite its fineness.

## Conclusions

It is possible to correct gingival asymmetry by performing dental preparation without finish line providing a correct periodontal analysis is first performed, which will contribute to successful soft tissue stabilization.
